# Teleosts as Models for Epigenetic Inheritance

**DOI:** 10.1111/mec.70292

**Published:** 2026-03-07

**Authors:** Adauto Lima Cardoso, Ozren Bogdanovic

**Affiliations:** ^1^ Centro Andaluz de Biología del Desarrollo CSIC‐Universidad Pablo de Olavide‐Junta de Andalucía Seville Spain; ^2^ Institute of Biosciences at Botucatu São Paulo State University Botucatu São Paulo Brazil; ^3^ Conselho Nacional de Desenvolvimento Científico e Tecnológico Brasília Brazil; ^4^ School of Biotechnology and Biomolecular Sciences University of New South Wales Sydney New South Wales Australia

## Abstract

Teleost fish exhibit a striking divergence from mammals in how they regulate their epigenome. Unlike mammals, they do not undergo the extensive DNA methylation erasure and resetting events that occur during early embryogenesis, germline specification, and spermatogenesis. Consequently, DNA methylation patterns in teleosts might persist across generations, making these species potentially valuable models for studying intergenerational and transgenerational epigenetic inheritance. Recent findings suggest that environmental perturbations can induce heritable epigenetic changes in teleosts, raising the possibility that, in the absence of global epigenetic reprogramming, such changes could have long‐lasting consequences. This review summarises current knowledge on DNA methylation stability and inheritance in teleosts, with a particular focus on the two teleost models, zebrafish and medaka. Moreover, the review discusses the ecological and evolutionary implications of epigenetic inheritance and highlights emerging experimental approaches for rigorously assessing transgenerational epigenetic effects in fish.

## 1 Introduction

Genetic information transmitted from parents to offspring forms the foundation of heritable traits across generations. However, numerous examples of inherited phenotypic variation in a wide range of organisms cannot be fully explained by classical Mendelian, sequence‐based inheritance. Beyond the DNA sequence itself, covalent chemical modifications of DNA, post‐translational modifications of histones, and various RNA species contributed by parental gametes can be transmitted to the next generation, providing a molecular basis for inter‐ and transgenerational epigenetic inheritance (TEI) (Boskovic and Rando 2018; Skvortsova et al. 2018). It is now well established that environmental factors, such as diet, exposure to chemicals, and stress, can reshape the epigenome, thereby creating a platform for the inheritance of epigenetic traits acquired during the parents' lifetime (Cavalli and Heard 2019).

Plants have played a central role in uncovering mechanisms of epigenetic inheritance, with maize paramutation being one of the classic examples, whereby one allele epigenetically silences its homologue in subsequent generations via small RNA pathways and chromatin remodelling complexes (Arteaga‐Vazquez and Chandler 2010). Another example is the methylation of the *Lcyc* gene promoter that governs floral symmetry variation in toadflax *Linaria vulgaris* (Cubas et al. 1999). On the other hand, non‐vertebrate models, *Caenorhabditis elegans* and *Drosophila melanogaster* stand out as key model organisms for TEI studies. These models are devoid of genomic DNA methylation (5mC, 5‐methylcytosine) and epigenetic inheritance occurs through mechanisms based on chromatin and small RNAs (Glastad et al. 2019; Rechavi and Lev 2017). In *C. elegans* , exposure to double‐stranded RNA (Ashe et al. 2012; Grishok et al. 2000) or environmental stressors (Rechavi et al. 2014) can induce heritable gene silencing that persists for multiple generations through small RNA amplification and nuclear Argonaute protein‐dependent mechanisms, establishing one of the best‐defined models of transgenerational epigenetic inheritance. In *D. melanogaster* , maternal deposition of piRNAs enables the intergenerational silencing of transposable elements and “paramutation‐like” phenomena at specific loci (Brennecke et al. 2008; de Vanssay et al. 2012), while Polycomb/Trithorax complexes can propagate repressive or active chromatin states through mitosis and even meiosis (Cavalli and Paro 1998; Ciabrelli et al. 2017). Besides *C. elegans* and *D. melanogaster* , which are completely devoid of 5mC, most non‐vertebrate lineages display very low levels of this modification (de Mendoza et al. 2019) and thus have not been widely considered as models for 5mC‐based TEI. Instead, it is likely that these organisms rely predominantly on chromatin‐ and small RNA‐based mechanisms to mediate heritable epigenetic information across generations.

In contrast to many non‐vertebrates or plants, epigenetic inheritance in mammals remains a matter of debate, largely because evidence is scarce and because mammalian development involves two rounds of genome‐wide reprogramming that erase and subsequently re‐establish DNA modification patterns, accompanied by extensive chromatin remodelling (Xu and Xie 2018). Nevertheless, some genomic regions, such as certain repeat classes, are not subjected to these reprogramming processes, as evidenced by the Agouti mouse models (Morgan et al. 1999). The Agouti mouse model illustrates how methylation of an intracisternal A particle (IAP) retrotransposon modulates *agouti* gene expression, influencing coat colour and metabolic phenotype in a heritable fashion (Cropley et al. 2006; Morgan et al. 1999). Moreover, recent advances in epigenome engineering have provided direct mechanistic evidence supporting the existence of epigenetic inheritance in mice. Experimental induction of locus‐specific 5mC in mammals has shown that such epigenetic modifications, once established, can persist through germline reprogramming and influence phenotypes across generations (Takahashi et al. 2023). Studies in mice have also demonstrated that environmentally induced metabolic states can be transmitted to offspring through small‐RNA‐ and chromatin‐based mechanisms, establishing a causal link between parental experience and heritable molecular memory (Argaw‐Denboba et al. 2024).

In contrast to mammals, multiple studies have shown that teleosts lack extensive DNA methylome reprogramming processes (Burgos‐Ruiz et al. 2025; Jiang et al. 2013; Kirchmaier et al. 2015; Ortega‐Recalde et al. 2019; Potok et al. 2013; Ross et al. 2023; Skvortsova et al. 2019), and therefore provide an experimental framework in which the persistence of inherited epigenetic states can be studied in the absence of large‐scale global epigenome erasure. In this context, teleost species such as *Danio rerio* (zebrafish) and *Oryzias latipes* (medaka) are emerging as particularly powerful vertebrate models for TEI studies, underpinned by their short generation spans, high‐quality genome assemblies, and extensive gene‐regulatory and epigenomic datasets (Baranasic et al. 2022; Bhandari 2016; Howe et al. 2013; Kirchmaier et al. 2015). Moreover, their phylogenetic position and ecological diversity provide opportunities to investigate the evolutionary context and potential adaptive significance of TEI. Thus, teleost models can facilitate research into inter‐ and transgenerational epigenetic transmission while providing critical insights into ecological and evolutionary implications of such processes.

This Mini Review summarises our current understanding of epigenetic inheritance in teleosts and highlights methodological advances enabling rigorous assessment of transgenerational epigenetic effects in this vertebrate group.

## 2 Zebrafish and Medaka as Teleost Models

Teleosts are considered highly relevant biological models because of several key experimental advantages. Notably, they exhibit short generation times and high fecundity, and their embryos are large, optically transparent, and develop externally, which are features that enable direct, non‐invasive observation of embryogenesis in real time. Teleost fishes encompass a diverse set of experimental systems spanning development, genetics, evolution, and aging. Among teleosts, zebrafish ( *Danio rerio* ) and medaka ( *Oryzias latipes* ) have become the most widely used models, supported by extensive genomic resources and versatile genetic tools (Howe et al. 2013; Kirchmaier et al. 2015). Other well‐established teleost models include stickleback ( *Gasterosteus aculeatus* ), widely used to study adaptation and speciation (Peichel and Marques 2017); the turquoise killifish ( *Nothobranchius furzeri* ), a powerful system for aging research (Valenzano et al. 2015); and the Mexican cavefish ( *Astyanax mexicanus* ), a key model in evolutionary developmental biology (Jeffery 2009).

Among all aforementioned models, zebrafish boasts the most comprehensive reference genome with detailed annotation and abundant regulatory information generated through coordinated efforts (Baranasic et al. 2022; Bradford et al. 2022). Combined with a vast repertoire of gene‐editing tools, transgenic lines, and established mutants, these resources greatly facilitate functional annotation (Simone et al. 2018). However, its genome architecture, shaped by a teleost‐specific whole‐genome duplication, introduces gene redundancies that can complicate single‐gene functional analyses (Taylor et al. 2003). Moreover, certain zebrafish lineages display high sensitivity to environmental fluctuations, requiring rigorous control of experimental conditions to ensure reproducibility (Bedell et al. 2025). At the same time, this intrinsic variability provides a unique opportunity to identify the genetic and epigenetic factors underlying lineage‐specific responses to environmental cues. Medaka, a teleost species separated from zebrafish by approximately 150–200 million years, has become an important complementary model. It possesses a compact, well‐annotated genome (~800 Mb) with lower repetitive content (Kasahara et al. 2007), a short generation time (Shima and Mitani 2004), and expanding genetic and genomic resources (Kirchmaier et al. 2015; Leger et al. 2022). Its wide natural diversity across geographic populations makes it ideal for studies of genetic variation, adaptation, and evolution (Hilgers and Schwarzer 2019). Although other teleosts such as cichlids, cyprinids, killifishes and salmonids exhibit even higher levels of ecological or morphological diversity, medaka stands out for combining natural variation with a high degree of experimental standardisation. Medaka also tolerates a broad range of environmental conditions, can be maintained at high densities, and, being phylogenetically distant from zebrafish, offers valuable opportunities for comparative vertebrate research (Shima and Mitani 2004). Overall, zebrafish and medaka provide complementary strengths—zebrafish with extensive genetic tools and medaka with broad evolutionary and environmental diversity—and provide two of the most versatile teleost models available today. Thus, studies based on these two models provide fundamental references that can be tested and expanded to non‐model species with greater diversity.

## 3 Epigenetic Inheritance Potential in Teleosts

Despite their genomic and phenotypic differences, both zebrafish and medaka constitute excellent models for vertebrate epigenetics research due to their high conservation of gene‐regulatory machinery with mammals. While chromatin marks in general are relatively dynamic and less stable across generations, DNA methylation, the covalent addition of a methyl group to the 5th carbon of the cytosine ring (5mC), represents one of the best‐studied and most robust epigenetic mechanisms (Mattei et al. 2022). 5mC predominantly occurs on the symmetrical CG dinucleotide therefore ensuring stable and faithful deposition and maintenance through its well‐described enzymatic machinery. Moreover, 5mC has the capacity to persist across generations while influencing diverse phenotypes, such as coat colour in mice (Morgan et al. 1999), division of labour in honeybees (Kucharski et al. 2008), or floral symmetry in the toadflax flower (Cubas et al. 1999). 5mC is thus a fundamental epigenetic mark that regulates gene expression and genomic stability, playing a major role in embryonic development, cellular differentiation, and the maintenance of cellular identity (Schubeler 2015). Nevertheless, despite its widespread presence in vertebrate genomes, direct regulatory activity appears to be confined to a limited subset of methylated loci.

**FIGURE 1:**
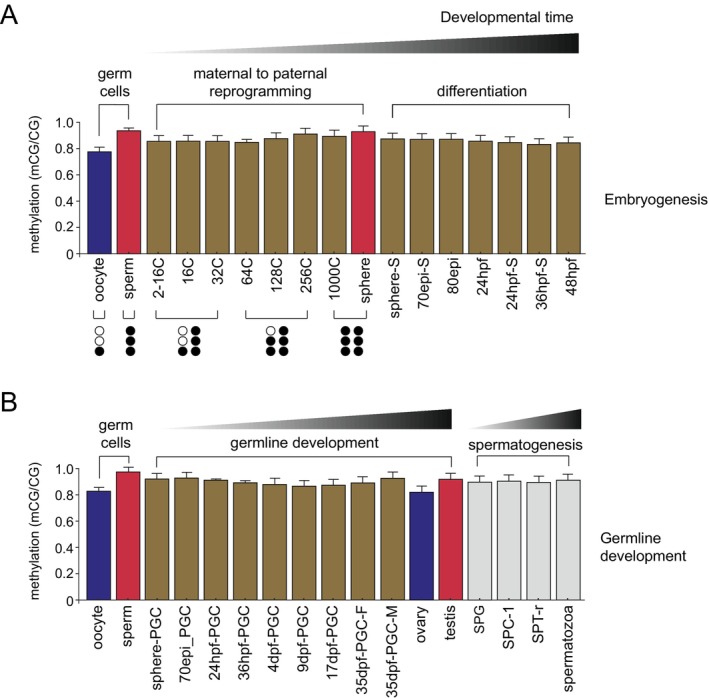
DNA methylation dynamics during the zebrafish life cycle. (A) Global CpG methylation (mCG/CG) values derived from published whole‐genome bisulfite sequencing studies of zebrafish embryogenesis (Bogdanovic et al. 2016; Jiang et al. 2013; Potok et al. 2013; Skvortsova et al. 2019). Sperm exhibits significantly higher 5mC content than the egg, and upon fertilisation the maternally inherited genome is remodelled to resemble the paternal one during zygotic genome activation (256‐cell to sphere stages). By the late blastula stage (sphere), the embryonic methylome becomes nearly identical to the sperm methylome, as indicated by the red colour. Later embryonic stages (70% epiboly to 48 hpf) do not undergo major changes in overall methylome composition. Black circles (methylated Cs) and white circles (unmethylated Cs) schematically illustrate that, in addition to global 5mC levels, methylation patterns are subtly remodelled in a maternal to paternal fashion, although no large‐scale reprogramming events occur. (C, cells; epi, epiboly; hpf, hours post‐fertilisation; S, somatic only). (B) CpG methylation dynamics during early germ cell development and adult gametogenesis. Profiles are shown for primordial germ cells (PGCs) collected at sphere, 70% epiboly, 24 hpf, 36 hpf, 4 dpf, 9 dpf, 17 dpf, and 35 dpf, as well as for ovary and testis (Jiang et al. 2013; Potok et al. 2013; Skvortsova et al. 2019; Wang et al. 2021). The only major loss of 5mC (~10%) is observed during female germline differentiation (ovary) (Wang et al. 2021). During spermatogenesis (SPC‐1, spermatocytes; SPG, spermatogonia; SPT‐r, round spermatids; and mature spermatozoa), no global 5mC changes have been reported (Burgos‐Ruiz et al. 2025). Error bars indicate the SEM calculated from replicated measurements originating from the same or different studies when available (min *n* = 2; max *n* = 3), otherwise, in case of a single replicate (*n* = 1), they represent the SEM of the full aggregated dataset encompassing all stages. 5mC values were extracted directly from the original publications based on authors reported data.

In teleost fishes, 5mC dynamics during early development present important differences when compared to eutherian mammals (Greenberg and Bourc'his 2019; Matlosz et al. 2024). Eutherian mammals undergo three rounds of genome‐wide reprogramming, consisting of near‐complete erasure and re‐establishment of 5mC marks in the preimplantation embryo (Smith and Meissner 2013), in primordial germ cells (PGCs) (Lee et al. 2014), and a somewhat milder reprogramming event that occurs during spermatogenesis, specifically at meiosis (Huang et al. 2023; Siebert‐Kuss et al. 2024). Whole‐genome bisulfite sequencing (WGBS), the gold‐standard method for 5mC analysis, has conclusively shown that teleosts do not reprogram their DNA methylome to the extent observed in mammals at any of these developmental stages or during any of these biological processes. In zebrafish, WGBS analyses have shown that sperm and oocyte genomes are highly methylated (~90% and ~80%, respectively) (Burgos‐Ruiz et al. 2025; Jiang et al. 2013; Murphy et al. 2018; Ortega‐Recalde et al. 2019; Potok et al. 2013; Skvortsova et al. 2019; Figure 1). Upon fertilisation, the early embryo adopts a paternal‐like methylome configuration by remodelling the maternal genomic contribution prior to the onset of zygotic genome activation (ZGA) (Jiang et al. 2013; Potok et al. 2013; Figure 1A). This remodelling event, however, does not involve any form of global epigenome erasure but instead consists of highly localised 5mC changes at defined loci. Moreover, during early development, 5mC acts as an epigenetic safe‐lock by repressing CG‐rich adult enhancers and adult tissue–specific genes (Wu et al. 2021). In contrast, embryonic enhancers are generally CG‐poor and escape 5mC–mediated repression, thus ensuring that global 5mC inheritance coupled with enhancer dememorization preserves embryonic transcriptional programs and enforces proper temporal gene expression. Consistent with these observations, the developing germline, consisting of primordial germ cells (PGCs), is also remodelled in a similar manner, without any notable global loss of 5mC (D'Orazio et al. 2021; Ortega‐Recalde et al. 2019; Skvortsova et al. 2019; Figure 1B). Similarly, WGBS analyses of sorted male germ cell populations, including primordial germ cells, spermatocytes, round spermatids, and mature sperm, failed to detect any major differences in 5mC patterning across these stages of spermatogenesis (Burgos‐Ruiz et al. 2025; Figure 1B).

In line with the slight differences observed in global 5mC levels between male and female adult germlines, the most pronounced reduction in 5mC during the zebrafish life cycle occurs during female germline formation, when global 5mC levels drop from ~90% to ~80% (Wang et al. 2021), a trend also seen across most somatic tissues profiled to date (Skvortsova et al. 2019). As with zebrafish, medaka also display maternal‐to‐paternal DNA methylome reprogramming, a somewhat higher 5mC content in sperm compared to eggs (~10%), and overall global lower 5mC levels in somatic tissues (Ross et al. 2023). These findings indicate that, despite their substantial evolutionary divergence, medaka and zebrafish undergo only minimal changes to their DNA methylomes across the life cycle. Supporting this notion, similar 5mC dynamics, consisting of maternal‐to‐paternal reprogramming, have been observed in the sea lamprey, a jawless vertebrate representing the sister lineage to all jawed vertebrates (Angeloni et al. 2024). Altogether, these findings argue that global 5mC reprogramming observed in eutherian mammals is likely a lineage‐specific innovation, whereas anamniotes are more likely to maintain and stably propagate their epigenome. This makes them potentially more vulnerable to environmental insults, but also valuable models for studies of epigenetic inheritance.

## 4 Evidence for Epigenetic Inheritance in Teleosts

Over the past decade, a growing body of work has demonstrated that teleost fishes can transmit environmentally induced epigenetic alterations, including changes in 5mC, across generations. Most of these studies have been conducted in zebrafish and medaka, whose prominence in TEI research reflects experimental tractability rather than any species‐specific propensity for TEI. In addition to studies of inherited epigenetic defects caused by genetic perturbations, such as maternally driven *hox* gene epimutations arising from chromatin‐modifier haploinsufficiency (Xue et al. 2022) or *dnmt1*‐dependent maintenance methylation defects persisting to the F3 generation (Iwanami et al. 2020), a diverse range of exposures has revealed inter‐ and transgenerational phenotypes, both molecular (e.g., DMRs) and morphological, which can be inherited through both maternal and paternal lineages (Table 1). Environmentally induced epigenetic alterations have been reported following exposure to phthalates and demethylating agents, with heritable locus‐specific 5mC changes detectable up to F2 (Kamstra et al. 2017). Additional studies have demonstrated transmission of molecular and phenotypic effects after parental exposure to mercury (Carvan 3rd et al. 2017), bisphenols (Gonzalez‐Rojo et al. 2019; Santangeli et al. 2019), insecticides (permethrin)—with behavioural alterations linked to gene‐regulatory disruption (Blanc et al. 2021)—and cadmium, which was associated with altered offspring sex ratios (Pierron et al. 2021). Recent work has further expanded the number of identified environmental stressors that can possibly cause heritable epigenetic effects. For example, microcystin‐LR, a naturally occurring toxin generated by cyanobacteria in warm aquatic environments, has been shown to alter behaviour and gene expression in offspring brains through paternal epigenetic alterations (Xu et al. 2023). Similarly, exposure to the flame retardant decabromodiphenyl ethane leads to transgenerational neurotoxicity passed through the paternal line (Sun et al. 2024), while arsenic exposure produces cognitive impairments that are inherited through both maternal and paternal germlines (Rachamalla, Carlos da Silva, et al. 2025). Although paternal methylomes generally appear more stable across generations (Figure 1), the global difference in 5mC levels between oocytes and sperm is in the order of ~10%. Given that this difference is relatively modest, it is highly plausible that environmentally induced epigenetic changes involving altered 5mC can serve as conduits for the intergenerational propagation of environmentally‐driven molecular perturbations through both maternal and paternal lines.

**TABLE 1:** Examples of epigenetic inheritance studies assessing DNA methylation changes in zebrafish.

Stressor	Study highlights and experimental design	Methodology	References
Arsenic	Sustained promoter hypermethylation of cognition‐related genes (*drd1*, *mao*, *bdnf*) across generations (F1‐F2)	qMSP	Rachamalla, da Silva Junior, et al. (2025)
Bisphenol A	Hypermethylation of *amh* promoter (F3) paralleled by reduction in active histone marks (F1, F2)	Bisulfite‐Pyrosequencing	Santangeli et al. (2019)
Bisphenol S	Fluctuations in global 5mC levels in ovaries and testes (F1, F2) upon parental exposure	ELISA	Hao et al. (2022)
Cadmium	Altered 5mC levels of *foxl2a* in females (F1‐F3)	Bisulfite‐Pyrosequencing	Pierron et al. (2021)
Cadmium	Transgenerational (F3) genetic and 5mC changes at the *cep19* locus	MeDIP‐Seq	Pierron et al. (2022)
Crude oil	Global hypomethylation (F1)	ELISA	Bautista et al. (2020)
Decabromodiphenyl ethane (DBDPE)	Locus‐specific (*crh*, *ugt1ab*) 5mC changes in F2	BS‐PCR	Sun et al. (2024)
Early high‐carbohydrate exposure	Stable hypomethylation of the *pck1* CpG island and reprogramming glucose metabolism in F0 and F1	BS‐BCR	Lu et al. (2022)
Ethylhexyl salicylate	Sex‐specific global 5mC abnormalities and inheritance of the paternal 5mC at *eya2*, *pcdh2g*5, and *pcdh2g1* genes	WGBS	Zhou et al. (2022)
Mercury	Transgenerational inheritance (F2) of novel DMRs associated with cellular signalling, metabolism and other terms	MeDIP‐Seq	Carvan 3rd et al. (2017)
Microcystin‐LR	Intergenerational inheritance (F1) of hypermethylated DMRs, upon paternal exposure	MeDIP‐Seq; RRBS	Zhao et al. (2021)
Microcystin‐LR	Hypomethylation of *dio3b* and *gad1b* promoters in F1 males, associated with neurotransmitter phenotypes	Bisulfite‐Pyrosequencing	Xu et al. (2023)
Mono(2‐ethylhexyl) phthalate and 5‐azacytidine	Transgenerational inheritance (F2) of newly acquired hypermethylated regions coinciding with regulatory elements	LC/MS; RRBS	Kamstra et al. (2017)
Permethrin	Locus‐specific 5mC changes linked to glutamatergic signalling and behavioural alterations (F2)	RRBS	Blanc et al. (2021)
Smchd1 haplo‐insufficiency	Intergenerational inheritance (F1) of aberrant 5mC states at *hoxc10a* locus	Bisulfite‐Pyrosequencing	Xue et al. (2022)
Viable hypomorphic allele of *dnmt1*	Transgenerational inheritance of aberrant 5mC patterning (F4) associated with impaired T‐cell development	WGBS	Iwanami et al. (2020)

Abbreviations: BS‐PCR, bisulfite sequencing polymerase chain reaction; DMRs, differentially methylated regions; ELISA, enzyme linked immunosorbent assay; LC/MS, liquid chromatography‐mass spectrometry; MeDIP‐Seq, methylated DNA immunoprecipitation sequencing; qMSP, quantitative methylation specific polymerase chain reaction; RRBS, reduced representation bisulfite sequencing; WGBS, whole genome bisulfite sequencing.

A growing body of research carried out over the last decade suggests that the paradigm of transgenerational epigenetic inheritance may extend beyond zebrafish to include medaka. For example, exposure to bisphenol A or 17α‐ethinylestradiol (EE2), both potent endocrine‐disrupting compounds, has induced transgenerational phenotypes of reproductive impairment and reduced embryonic survival in these fish (Bhandari et al. 2015). In a similar vein, early‐life parental exposure to the synthetic herbicide atrazine resulted in reproductive disruption in unexposed F2 progeny, which involved altered testicular histology, reduced sperm motility, and persistent changes in the expression of genes involved in steroidogenesis and 5mC maintenance (Cleary et al. 2019). A more recent experiment using marine medaka (*O. melastigma*) demonstrated that developmental exposure to fenvalerate and sulfamethoxazole, two common environmental pollutants, triggered persistent transcriptomic disruptions in juvenile fish, particularly in pathways related to cell cycle, fatty acid metabolism, and steroid biosynthesis, with potential for transgenerational propagation (Chen et al. 2025). Although these studies indicate potential epigenetic inheritance mediated by 5mC in medaka, detailed molecular studies using more refined methods are still needed to provide more robust and conclusive data.

Beyond zebrafish and medaka, intergenerational and transgenerational epigenetic inheritance have now been documented across a diverse array of teleost species. These studies collectively demonstrate that naturally occurring, environmentally induced DNA methylome alterations (Shao et al. 2014; Vernaz et al. 2022, 2021), as well as those arising from captivity or husbandry practices (Rodriguez Barreto et al. 2019; Venney et al. 2025), or thermal variations (Brionne et al. 2023; Sanchez‐Baizan et al. 2025; Valdivieso et al. 2023) can persist across generations. Overall, these studies underscore that teleosts exhibit reproducible intergenerational and, in some cases, bona fide transgenerational inheritance of environmentally induced epigenetic and phenotypic modifications, although the underlying molecular mechanisms, as well as their implications for adaptability to diverse environmental factors, remain unresolved.

## 5 Common Limitations of Epigenetic Inheritance Studies in Teleosts

The diversity of species, inducing agents, and methodological approaches among studies (Table 1), combined with evidence of limited global 5mC reprogramming during early embryogenesis, germline specification, and spermatogenesis, points to 5mC as a potentially environmentally sensitive mark whose incomplete reprogramming in teleosts enables its persistence across generations, potentially contributing to adaptive plasticity as well as susceptibility to environmental stressors. Despite these important advances in the field, several limitations stand out. The first is methodological heterogeneity: while some studies adopt comprehensive base‐resolution sequencing approaches (e.g., WGBS), others rely on targeted or lower‐resolution methods (e.g., ELISA, qMSP, BS‐PCR). Although more accessible, these latter techniques may under‐ or overestimate epigenetic variation, limiting the identification of consistent patterns (Harris et al. 2010). In this context, ELISA‐based measurements can be highly misleading in early development, particularly in organisms with large eggs (Matlosz et al. 2024). The high copy number of largely unmethylated mitochondrial DNA can vastly exceed nuclear genomic content, artificially diluting apparent 5mC levels in such bulk assays (Ross et al. 2023). Another critical limitation is the lack of independent replication, with many studies based on a single lineage or population without validating whether patterns are reproducible across genetic backgrounds or ecological settings. A further concern relates to the evolutionary interpretation of transgenerational epigenetic changes. Adaptive advantages of inherited epigenomic modifications are more plausible and have indeed been demonstrated in short‐lived species and in controlled environments, where rapid phenotypic responses can improve fitness across generations (Perez and Lehner 2019). However, for longer‐lived, mobile organisms such as many fish and other vertebrates, the evolutionary benefit of persistent epigenetic inheritance is less clear, and evidence supporting such adaptive roles is limited.

More importantly, the field still lacks a mechanistic understanding of how environmentally induced or inherited epigenetic changes produce stable, functional outcomes over time (Arzate‐Mejia and Mansuy 2022). Additionally, many studies display a shortage of sequencing controls that can clearly distinguish between genetic and epigenetic sources of variation. For example, within the specific context of epigenetic inheritance in teleosts, cadmium‐induced 5mC changes that persisted to F3 were ultimately traceable to a nearby single nucleotide polymorphism (SNP) that created or removed a CpG site and strongly modulated 5mC at adjacent loci (Pierron et al. 2022). Although feasible, studies often do not implement designs that control for underlying genetic differences (Heyn et al. 2013), such as using different strains, reciprocal crosses, or high‐resolution long‐read sequencing combined with DNA methylome profiling. Moreover, while whole‐genome sequencing (WGS) is undoubtedly useful for characterising underlying genetic variation among individuals, populations, or experimental lines, it is often omitted from epigenetic studies, and even when included, short‐read WGS alone cannot reliably disentangle genetic variation from epigenetic modifications, particularly in non‐model organisms with complex or poorly resolved genomes. These technical limitations complicate efforts to attribute observed phenotypic changes specifically to epigenetic mechanisms. Thus, although evidence for transgenerational epigenetic inheritance is steadily accumulating in teleosts, the field still struggles with standardising methods, ruling out genetic confounders, and connecting epigenetic variation to fitness‐related traits in realistic ecological contexts.

## 6 Future Directions and Opportunities

Research on epigenetic inheritance in teleosts has advanced considerably in recent years, driven by innovations in molecular biology and sequencing technologies. Emerging tools, such as long‐read sequencing, single‐cell epigenomics, and genome editing, are expanding the resolution at which 5mC and other epigenetic marks can be studied. Long‐read platforms (e.g., PacBio HiFi, Oxford Nanopore) enable the simultaneous detection of genetic and epigenetic variation at the single‐molecule level, improving our ability to characterise methylation landscapes in repetitive regions and structural variants, which are often underexplored with short‐read methods (Liu and Conesa 2025). From an ecological and evolutionary perspective, teleosts provide a remarkable model to investigate how environmental stressors induce heritable epigenetic changes. Experimental exposures to pollutants and climate stressors have already demonstrated that methylome changes can persist across generations. These findings also have broader ecological implications. If environmentally induced epigenetic states are stably inherited (Vernaz et al. 2021), they may contribute to population resilience or vulnerability in rapidly changing ecosystems (Heckwolf et al. 2021; Rodriguez Barreto et al. 2019). In this context, studying teleosts within an epigenetic inheritance framework can provide valuable insights into how biodiversity responds to anthropogenic pressures and whether epigenetic plasticity constitutes a buffer against environmental degradation (Abdelnour et al. 2024).

While 5mC has been the primary focus of this review, it is important to note that additional epigenetic mechanisms, including histone modifications, histone variant dynamics, and non‐coding RNAs, also contribute to developmental regulation and phenotypic inheritance in teleosts. These complementary mechanisms, their evolutionary diversification in duplicated teleost genomes, and their roles in early embryogenesis and physiology have been reviewed elsewhere (Best et al. 2018). Notwithstanding the diversity of epigenetic marks involved in TEI, the field faces persistent challenges related to experimental design and methodological rigor. Standardising protocols remains a key priority, as heterogeneous approaches limit comparability across studies. Experimental designs that include controlled crosses, clearly defined environmental treatments, and systematic monitoring across multiple generations are essential to distinguish heritable epigenetic changes from transient responses induced by the environment. In this context, conducting joint analysis of phenotypes and epigenetic marks is important to directly test if specific epigenetic variations are associated with adaptive phenotypes, an issue which remains unresolved to date (Angers et al. 2020; Kelley et al. 2021; Uller et al. 2013). Future studies should incorporate well defined sequencing controls, account for confounding factors such as density or diet, and implement independent replication across genetic backgrounds and environmental contexts (Dura et al. 2025). To minimise confounding effects, future studies should aim to combine WGBS and WGS where possible to clearly separate epigenetic variation from underlying genetic differences. Adoption of long‐read sequencing technologies is also recommended, as such approaches can provide simultaneous genome and DNA modification profiling from the same sample in a highly quantitative and well‐controlled manner. Moreover, large‐scale, multigenerational studies conducted both in controlled laboratory settings and in semi‐natural environments will be essential for assessing the stability, persistence, and adaptive potential of epigenetic marks, whereas comparative work across species from different taxonomic groups may further clarify the contribution of epigenetic inheritance to biodiversity and responses to environmental variation (Robaire et al. 2022). Ultimately, we believe that combining mechanistic studies with ecological monitoring could position teleosts as sentinel organisms for environmental change, linking molecular processes to ecosystem‐level responses and enabling a more rigorous evaluation of the evolutionary significance of epigenetic inheritance in natural populations.

## Author Contributions

O.B. conceived the study. O.B. and A.L.C. wrote and edited the manuscript.

## Funding

This work was supported by the Ministerio de Ciencia, Innovación y Universidades/Agencia Estatal de Investigación (MICIU/AEI, https://doi.org/10.13039/501100011033) through projects PID2021‐128358NA‐I00 (co‐funded by ERDF/EU), and CNS2023‐144039 (co‐funded by NextGenerationEU/PRTR) to O.B.

## Conflicts of Interest

The authors declare no conflicts of interest.

## Data Availability

Data sharing not applicable to this article as no datasets were generated or analysed during the current study.
